# Genomic and hyperspectral imaging-based prediction blending enables selection for reduced deoxynivalenol content in wheat grains

**DOI:** 10.1093/g3journal/jkaf176

**Published:** 2025-08-06

**Authors:** Jonathan S Concepcion, Amanda D Noble, Addie M Thompson, Yanhong Dong, Eric L Olson

**Affiliations:** Department of Plant, Soil, and Microbial Sciences, Michigan State University, East Lansing, MI 48823, United States; Department of Plant, Soil, and Microbial Sciences, Michigan State University, East Lansing, MI 48823, United States; Department of Plant, Soil, and Microbial Sciences, Michigan State University, East Lansing, MI 48823, United States; Department of Plant Pathology, University of Minnesota, St. Paul, MN 55108, United States; Department of Plant, Soil, and Microbial Sciences, Michigan State University, East Lansing, MI 48823, United States

**Keywords:** hyperspectral imaging, deoxynivalenol, wheat, genomic selection, phenomic selection

## Abstract

Breeding for low deoxynivalenol (DON) mycotoxin content in wheat is challenging due to the complexity of the trait and phenotyping limitations. Since phenomic prediction relies on nonadditive effects and genomic prediction on additive effects, their complementarity can improve selection accuracy. In this study DON-infected wheat kernels were imaged using a hyperspectral camera to generate reflectance values across the spectrum of visible and near-infrared light that were used in phenomic predictions. Five Bayesian generalized linear regression models and 2 machine learning models were trained using phenomic and genomic predictions from advanced soft winter wheat breeding lines evaluated in 2021 and 2022. Across all training sets and models, phenomic predictions using wavebands in the visible light spectrum (400 to 700 nm) had higher predictive ability than genomic predictions or phenomic predictions using the full waveband range (400 to 1,000 nm). Forward prediction using 2021 trial, 2022 trial, and combined trials as the training set was performed using model blending on 2 sets of F_4:5_ selection candidates evaluated independently in 2022 and 2023. The phenotypic and genetic correlations, as well as indirect selection accuracies, of the model averages of phenomic predictions and combined phenomic and genomic predictions were higher than genomic predictions alone. Accuracies depended on the combination of training set and selection candidates. Unsupervised K-means clustering using the blended predicted values partitioned selection candidates into 2 groups with high and low mean observed DON content. This study demonstrates the potential of hyperspectral imaging-based phenomic prediction to complement genomic prediction and highlights considerations for prediction-based selection of low DON in wheat.

## Introduction

Breeding for complex traits like reduced deoxynivalenol (DON) mycotoxin accumulation, known as type III resistance to Fusarium head blight (FHB), presents a significant challenge in wheat breeding (Bai et al. 2018). DON is commonly measured using gas chromatography/mass spectrometry (GC/MS) (Tacke and Casper 1996), but this method is often time-consuming and labor-intensive due to the nature of sample preparation (Steiner et al. 2017). For breeding programs that outsource GC/MS analysis, the time required to generate DON data can extend the time frames for selection, potentially impacting decision making and the length of breeding cycles. To address these limitations, researchers have explored alternative approaches for DON measurement to reduce reliance on GC/MS (Buerstmayr et al. 2020; Alisaac and Mahlein 2023).

Since its introduction, genomic prediction has been used extensively to improve complex traits (Haile et al. 2021 ; Shahi et al. 2022), including FHB resistance (Rutkoski et al. 2012), with moderate to high prediction accuracies. Genomic prediction enables the estimation of an individual’s genetic breeding value based on genomic information, often using single nucleotide polymorphisms (SNPs) (Heffner et al. 2009). One advantage of genomic prediction is enabling early and accurate predictions, thereby accelerating the breeding process and increasing selection efficiency, allowing breeders to evaluate a larger number of individuals, and increasing selection intensity (Meuwissen et al. 2001; Heffner et al. 2009; Crossa et al. 2014). However, environmental effects and genotype-by-environment interactions still need to be considered for the target trait, which remains a weakness of genomic prediction, especially when multiple years and environments are considered (Jackson et al. 2023). While costs per sample have decreased significantly, genotyping large numbers of individuals still requires substantial financial resources. For breeding programs conducting in-house sampling and DNA extraction, this process can also be time-consuming and labor-intensive.

In recent years, phenomic selection has emerged as a potential alternative to genomic prediction, addressing some of its practical challenges, particularly in terms of implementation (Rincent et al. 2018). The primary advantage of phenomic selection lies in its ability to achieve a comparable, and in some cases higher, level of accuracy than genomic prediction (Rincent et al. 2018). Phenomic selection uses high-throughput phenotyping tools, such as near-infrared spectroscopy, to capture detailed phenomic data that reflect the physical and biochemical characteristics of the sample (Rincent et al. 2018). In the case of FHB resistance, several studies have demonstrated the potential application of image-based phenomic platforms, for FHB evaluation (Alisaac et al. 2018; Mahlein et al. 2019; Liang et al. 2024). Despite these advancements, phenomic prediction for DON, specifically in comparison with genomic prediction, has not been extensively explored, leaving room for further research to leverage its full potential in breeding programs targeting DON reduction. Wang et al. (2025) argued, however, that the comparison of accuracy (by phenotypic correlation between actual and predicted values) between phenomic and genomic prediction could be misleading. Therefore, using prediction accuracy (Wang et al. 2023) and indirect selection accuracy (Lopez-Cruz et al. 2025), which considers genetic correlations and heritability, shall provide better and valuable comparison between both prediction approaches.

Recently, efforts have been made to integrate information from varied phenotyping platforms to predict and select for traits in breeding programs (Märtens et al. 2016; Rutkoski et al. 2016; Crain et al. 2018; Galán et al. 2020; Adak et al. 2023; Roth et al. 2023; Togninalli et al. 2023), with many using drone-based platforms to evaluate agronomic traits. Thapa et al. (2024) recently demonstrated the potential integration of hyperspectral imaging with genomic information to predict FHB-related traits, including DON accumulation, using deep learning approaches. Their results demonstrated significant improvements in prediction accuracy compared to genomic prediction alone. Model ensembling and blending approaches, such as Bayesian model averaging, weighted model averaging, and least squares model averaging, have long been used in fields outside of plant breeding (Raftery et al. 1997; Wasserman 2000; Hansen 2007; Magnus et al. 2010). An important contribution to the application of model ensembling in plant breeding was made by Kick and Washburn (2023), revealing that combining linear and nonlinear models using genomic, environmental, and crop management data provided better accuracy than base (or individual) models for grain yield.

This study explores the use of hyperspectral imaging-derived spectral reflectance values in the phenomic prediction of DON mycotoxin content in soft winter wheat grains and how phenomic prediction could complement genomic prediction. Simple model averaging was explored as a model blending approach to integrate both types of predictions. The utility of phenomic, genomic, and blending of both phenomic and genomic predictions models was explored by forward prediction in a separate set of F_4:5_-derived selection candidates. Model blending by unsupervised clustering of genotypes based on predicted DON values from various models was explored in a breeding selection scheme.

## Materials and methods

### Plant materials for training population and field establishment

A total of 558 soft winter wheat genotypes (430 soft red and 128 soft white) comprised of advanced breeding lines and commercially released varieties were evaluated for DON mycotoxin content in response to FHB in 2021 (n = 307) and 2022 (n = 288). The 558 genotypes served as training populations in subsequent analysis. A set of 37 genotypes were evaluated in both years. Limited overlap between years is due to the use of advanced breeding lines from an active breeding program where new entries are cycled through testing stages each year. The soft white winter wheat genotype, MI14W0190, was the FHB-resistant, low DON check. The soft white winter wheat variety, Ambassador, was the FHB-susceptible, high DON checks. In both years, genotypes were evaluated in a misted and inoculated *Fusarium* screening nursery in East Lansing, Michigan, using a completely randomized design with 2 replicates.

### *Fusarium inoculum* preparation and field inoculation

*Fusarium graminearum* cultures were collected in 2020 from Huron, Ingham, Monroe, Tuscola, and Sanilac counties and in 2021 from Huron, Ingham, Monroe, and Saginaw counties in Michigan, United States, for the 2021 and 2022 trials, respectively. Isolates were grown by placing infected seeds in Nash–Snyder media for 5 to 7 d at room temperature. Approximately 1.5 kg of corn kernels were soaked in deionized water for 24 to 48 h, placed in spawn bags with 0.2-μm filter patch (Unicorn Bags, TX, United States) and autoclaved 3 times for 90 min. A 4-to 6-day-old culture and 100 mL autoclaved deionized water were added to each autoclaved spawn bag. Isolates from different locations were cultured separately, and after approximately 2 weeks, infected grain spawns were dried in a biohazard hood at ambient temperature. The *F. graminearum* grain spawn cultures were pooled in equal proportions by weight prior to inoculation. Field inoculation began 5 weeks before flowering and grain spawn was applied 4 to 5 times. To promote proper condition for infection and disease development, a misting system was run throughout the nursery 10 min every hour for 12 h, 6:00 AM to 6:00 PM.

### DON measurement

A sample of infected heads was collected from the middle 0.3 m of each replicate of each genotype in the *Fusarium* screening nursery. A 10-g subsample of infected wheat kernels was ball-milled using Restch MM 400 miller (Retsch, PA, United States). The flour samples were then sent to the Department of Plant Pathology, University of Minnesota, for DON concentration measurement using GC/MS.

### Hyperspectral imaging and processing

A sample of 50 to 80 wheat kernels from each replicate of each genotype were placed against a dark background side-by-side a white reference panel and were imaged using a mobile, handheld hyperspectral camera, Specim IQ (Specim, Oulo, Finland). Imaging was done inside a 20 × 20 × 20 inch light box (Finnhomy, United States) using the attached light-emitting diode (LED) light source with the camera mounted on a tripod and angled 45° facing downward over the kernels and the focus set at automatic. To capture the raw reflectance values from 204 wavebands (397 to 1004 nm), the camera was set at Default Recording Mode with an integration time of 30 to 40 s. Hyperspectral image files (.dat) were stored in Specim IQ studio and imported as raster layer to QGIS 3.10.2 (QGIS 2020) for processing. Multiband color was used for rendering, with band 088 (651.92 nm) as Red Band, band 057 (560.30 nm) as Green Band, and band 037 (501.72) as Blue Band. Color enhancements were set at Stretch to MinMax and normal blending mode. Raster calculated images at a 0.3 to 0.8 threshold were saved as GeoTIFF (.tif) file and converted to vector image (Polygonize) using default settings. Unnecessary features were removed using toggle edit to determine the region of interest (wheat kernels) and saved as ESRI shape file (vectorized image) (.shp). From the ESRI shape file, spectral reflectance values in each waveband were extracted using “raster” package (Hijmans 2010) in R v4.2.2 (R Core Team 2023). A Savitzky–Golay filter was used for smoothing the generated spectral reflectance values across wavebands at a window size of 11 and polynomial degree of 3 (3rd degree) using the “signal” package in R (Ligges et al. 2006).

### Statistical analysis

ANOVA F-tests were carried out to assess the variation in DON content and spectral reflectance values among the genotypes tested in each year. Best linear unbiased estimates (BLUEs) of DON content and spectral reflectance values in all wavebands generated for all genotypes in each year were generated using “lsmeans” package (Length 2016) following the model


(1) $$Y_{ij}=\mu +G_{i}+\varepsilon_{ij}$$

where *Y_ij_* is the response of the *i*th genotype in the *j*th observation, µ is the overall mean, *G_i_* is the fixed effect of the *i*th genotype, and $\varepsilon_{ij}$ is the residual error for the *i*th genotype in the *j*th observation.

BLUEs of DON content and spectral reflectance values for all wavebands across years were modeled using “lme4” package in R (Bates et al. 2015) following the model


(2) $$Y_{ijk}=\mu +G_{i}+T_{j}+(GT{)}_{ij}+\varepsilon_{ijk}$$

where *Y_ijk_* is the response of the *i*th genotype in the *j*th year for the *k*th observation, µ is the overall mean across genotypes and years, *G_i_* is the fixed effect of the *i*th genotype, *T_j_* is the random effect of *j*th trial, *(GT)_ij_* is the random effect of the interaction between the *i*th genotype and the *j*th trial, and $\varepsilon_{ijk}$ is the residual error. Mean DON content and spectral reflectance values across years were computed using “lsmeans” package (Lenth 2016) in R after modeling. Pearson's correlation coefficient was used to assess the relationship between BLUEs of DON content and BLUEs of reflectance values at all individual wavebands within and across years.

### Heritability estimation

Heritability for DON content based on entry mean within years was calculated based on the following equation:


(3) $$H_{Geno}^{2}=\;\frac{\sigma_{G}^{2}}{\sigma_{G}^{2}+(\sigma_{e}^{2}/r)}$$

where $H_{Geno}^{2}$ is the estimated heritability on an entry mean basis, *r* is the number of replicates per genotype (entry), $\sigma_{e}^{2}$ is the error variance (residual mean square), and $\sigma_{G}^{2}$ is the genotype (entry) variance calculated following the equation:

Heritability based on entry mean across years was calculated based on the following equation:


(4) $$H_{GenoT}^{2}=\;\frac{\sigma_{G}^{2}}{\sigma_{G}^{2}+(\sigma_{GT}^{2}/r)+(\sigma_{e}^{2}/ry)}$$

where $H_{GenoT}^{2}$ is the estimated heritability on an entry mean basis, *r* is the number of replicates per genotype (entry), *T* is the number of trials, $\sigma_{e}^{2}$ is the error variance (residual mean square), and $\sigma_{G}^{2}$ is the genotype (entry) variance, and $\sigma_{GT}^{2}$ is the genotype by trial variance.

### DNA isolation and SNPs genotyping

Tissue was collected from all genotypes evaluated and DNA was isolated according to Wiersma et al. (2016). Genotyping-by-sequencing libraries were prepared according to Poland et al. (2012) scaled to a 24 μL volume in 384-well format. Libraries were sequenced at 384-plex on an Illumina HiSeq 4000 instrument. SNPs were called using the TASSEL 5 GBS pipeline (Glaubitz et al. 2014). Reads were aligned to the RefSeq v2.0 wheat reference genome assembly (International Wheat Genome Sequencing Consortium) using default parameters. For the GBSSeqToTagDBPlugin and ProductionSNPCallerPluginV2 steps, the k-mer length was set to 64 base pairs and a minimum coverage of 5 reads was required for each k-mer. Default settings were used for all other steps. SNPs were initially called using all families and parents. SNPs were subsequently filtered for 0.70 call rate and 0.05 minor allele frequency. A total of 15,456 SNPs out of 386,651 SNPs remained after filtering.

### Univariate genomic and phenomic prediction model assessment

Two overlapping sets of hyperspectral bands were used in phenomic predictions of DON content, all 204 wavebands from 400 to 1,000 nm in the visible light and near-infrared spectrum (VIS/NIR) and wavebands in the VIS region from 400 to 800 nm. Genomic predictions were made using 15,456 SNPs across the wheat genome. Both phenomic and genomic prediction for DON was assessed using Bayes A, Bayes B, Bayes C, Bayesian Ridge Regression (BRR), and Bayesian Least Absolute Shrinkage and Selection Operator (LASSO) (BayesL) with 50,000 iterations and BurnIn set at 5,000, implemented in the “BGLR” package (Pérez and de los Campos 2014). Ridge Regression Best Linear Unbiased Prediction (RRBLUP) was executed using “rrBLUP” package in R (Endelman 2011). Extreme Gradient Boosting (XGBoost) was implemented using the “xgboost” package (Chen and Guestrin 2016) with parameters eta (learning rate) = 0.01, gamma = 0.2, max_depth (maximum tree depth) = 6, min_child_weight (minimum sum of instance weights) = 0.20, subsample = 1, and colsample_bytree (fraction of features (columns) used to grow each tree) = 1. Random Forest was executed using the “caret” package (Kuhn 2008) with default parameters (ntree = 500, nodesize = 5, maxnodes = unlimited). A total of 100 individual 5-fold cross-validations were carried out with 80% of the genotypes designated in the training set for all the models. Predictive ability of phenomic and genomic prediction for all models were computed as the average Pearson's correlation (predictive ability) between the actual DON content and predicted values from all 100 cycles in each model.

Genetic correlation as the Pearson correlation coefficient between the marker (or waveband for phenomic prediction)-based estimated breeding values (EBVs) derived from each models tested and the EBVs obtained from best linear unbiased prediction (BLUP) using the realized genomic relationship matrix (G). The realized G matrix was computed as the cross-product of standardized marker data, and mixed model solutions were obtained using the “mixed.solve” function in the “rrBLUP” package (Endelman 2011). From here, the genetic variance of the predicted and actual DON content, and its corresponding covariance was used to calculate the genetic correlation following the formula


(5) $$r_{g}=\;\frac{Cov_{XI}}{\sqrt{\left(Var_{X})\;(Var_{I}\right)}}$$

where *Cov_XI_* is the covariance between the observed DON content (*X*) and the predicted DON content (*I*), *Var_X_* is the genetic variance of the observed DON content, and *Var_I_* is the genetic variance of the predicted DON content.

Prediction accuracy (*Acc_(A)_*) was also calculated from each cross-validation cycle following the formula adapted from Wang et al. (2023):


(6) $$Acc_{\left(A\right)}=\;r_{G}\sqrt{H_{A}^{2}}$$

where *r_G_* is the genetic correlation between predicted and actual DON content, and $H_{A}^{2}$ is the estimated heritability of actual DON content in the testing set calculated by dividing the estimated genetic variance of the actual DON content in the testing set ($\sigma_{G\_X}^{2}$) by its estimated phenotypic variance ($\sigma_{P\_X}^{2}$).

All cross-validations, model fitting, heritability estimates, phenotypic and genotypic correlations, and prediction accuracy assessment were carried out in R v4.2.2 (R Core Team 2023).

### Feature selection

The most informative wavebands were identified for each of the 2021 and 2022 trials and combined trials (across years) using recursive partitioning and regressive tree model with the “caret” package (Kuhn 2008) carried out in R v4.2.2 (R Core Team 2023). Feature importance score (scaled relative importance percentage) of 100 or closer means higher association with DON content, whereas scores of 0 or closer means no or less association with DON.

### Forward prediction

To test the predictive ability of the trained models, a set of 239 F_4:5_ generation breeding lines evaluated in 2022 and 217 F_4:5_ breeding lines evaluated in 2023 were used as prediction sets of selection candidates. Field establishment, field inoculation, DON measurement, and hyperspectral imaging and processing of selection candidates followed the procedures described for the 2021 and 2022 training sets. To simulate actual breeding scenarios, models trained using 2021 and 2022 trials, and BLUEs generated across years were used to predict the DON content of selection candidates. Forward predictions were carried out separately for phenomic and genomic predictions using all the models tested and used for cross-validation.

### Evaluation of blended predictions using trained models

Three approaches of model blending using all the models tested were investigated: (i) phenomic predictions of DON content, (ii) genomic predictions of DON content, and (iii) both phenomic and genomic predictions of DON content. Blended forward prediction by model averaging was carried out following the formula


(7) $$\hat{y}=\frac{1}{M}\overset{M}{\underset{m=1}{\sum }}\;{\hat{y}}_{m}$$

where *ŷ* is the blended (averaged) forward prediction, *M* is the total number of individual prediction models, and *ŷ_m_* is the predicted value from the *m*th model.

Phenotypic correlation between predicted and actual DON content in the selection candidates were carried out using Pearson's correlation. Genetic correlation (*r_G_*) and prediction accuracy (*Acc_A_*) was also determined following the approach and formula adapted from (5 and 6), respectively.

In addition, indirect selection accuracy, *Acc_(I)_*, of the blended predictions was determined based on the formula adapted from Lopez-Cruz et al. (2020):


(8) $$Acc_{\left(I\right)}=H_{I}(cor_{XI})$$

where *cor_XI_* is the genetic correlation between the observed DON content of the selection candidates (*X*) and the predicted DON content of the selection candidates based on blended predictions (*I*), and *H_I_* is the square root of the estimated broad-sense heritability ($H_{I}^{2}$) in the predicted DON content of the selection candidates based on blended predictions, where $H_{I}^{2}$ is the estimated heritability of the blended DON content predictions of the selection candidates calculated by dividing the estimated genetic variance of the blended DON content predictions of the selection candidates ($\sigma_{G\_I}^{2}$) by its estimated phenotypic variance ($\sigma_{P\_I}^{2}$).

### Blended clustering for selection DON content selection

Unsupervised K-means clustering was carried out using the “cluster” package in R (Maechler 2018) in selection candidates using 3 different blending of 8 phenomic, 8 genomic, and 16 phenomic and genomic prediction models. Clustering was carried out based on the objective function:


(9) $$J\;=\;\overset{K}{\underset{k=1}{\sum }}\;\sum_{i\in C_{k}}∥{\hat{y}}_{i}-\mu_{k}{∥}^{2}$$

where *J* is the within-cluster sum of squares, *K* is the predefined number of clusters, *C_k_* is the set of data points assigned to cluster *k*, *ŷ_i_* = [*ŷ_i1_*, *ŷ_i2_, …, ŷ_iM_*] is the vector of predicted values from *M* different models for data point *i*, and µ*_k_* is the centroid of cluster *k*, defined as the mean vector of predicted values across all points in *C_k_*. Two clusters were used for clustering (centers = 2) as per result of Silhouette method employed to identify the optimum number of clusters. Two clusters were used for all K-means clustering runs for consistency.

## Results

### DON evaluation

**Fig. 1.**
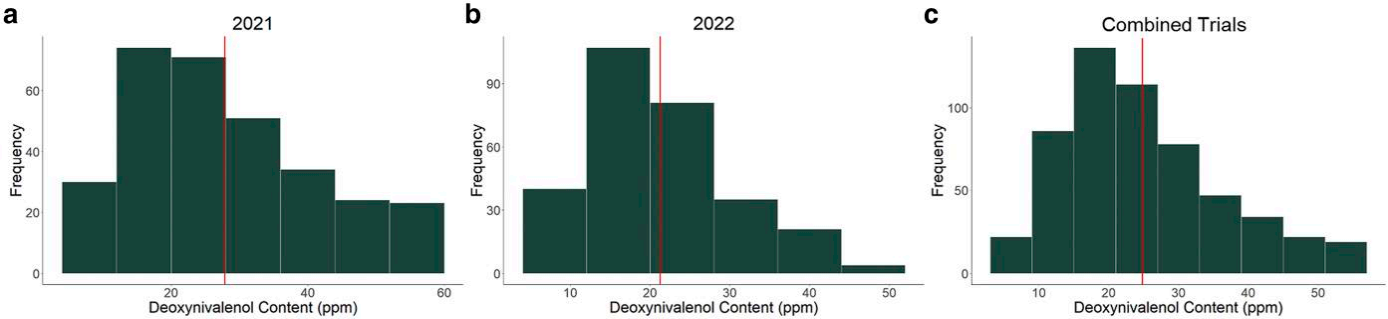
Frequency distributions of DON content in genotypes tested in (a) 2021, (b) 2022, and (c) 2021 and 2022 combined. Solid bar represents average DON content among the genotypes tested for individual trials and across years. MI14W0190 and Ambassador, resistant and susceptible checks, respectively, were also highlighted.

Significant variation in DON content was observed among the genotypes (*P* < 0.00001) tested in 2021 and in 2022 (Fig. 1). In 2021, 229 genotypes (74.6%) had significantly lower DON content than the susceptible check Ambassador, while 194 genotypes (63.5%) were not different from the resistant check MI14W0190, and 27 (8.8%) had significantly lower DON content than MI14W0190. In 2022, 279 genotypes (96.9%) had significantly lower DON content than Ambassador, and 194 (67.4%) demonstrated no difference from MI14W0190. No genotypes in 2022 had lower DON content than MI14W0190. Across both years, there was significant variation in DON due to genotype (*P* < 0.00001) (Fig. 1), year (*P* = 0.0000756), and genotype-by-year interaction (*P* = 0.000174). The entry mean heritability for DON across years at 0.55 was lower compared to single-year estimates for 2021 at 0.85 and 2022 at 0.81 (Supplementary Table 1).

### Hyperspectral imaging of DON-infected wheat kernels

**Fig. 2.**
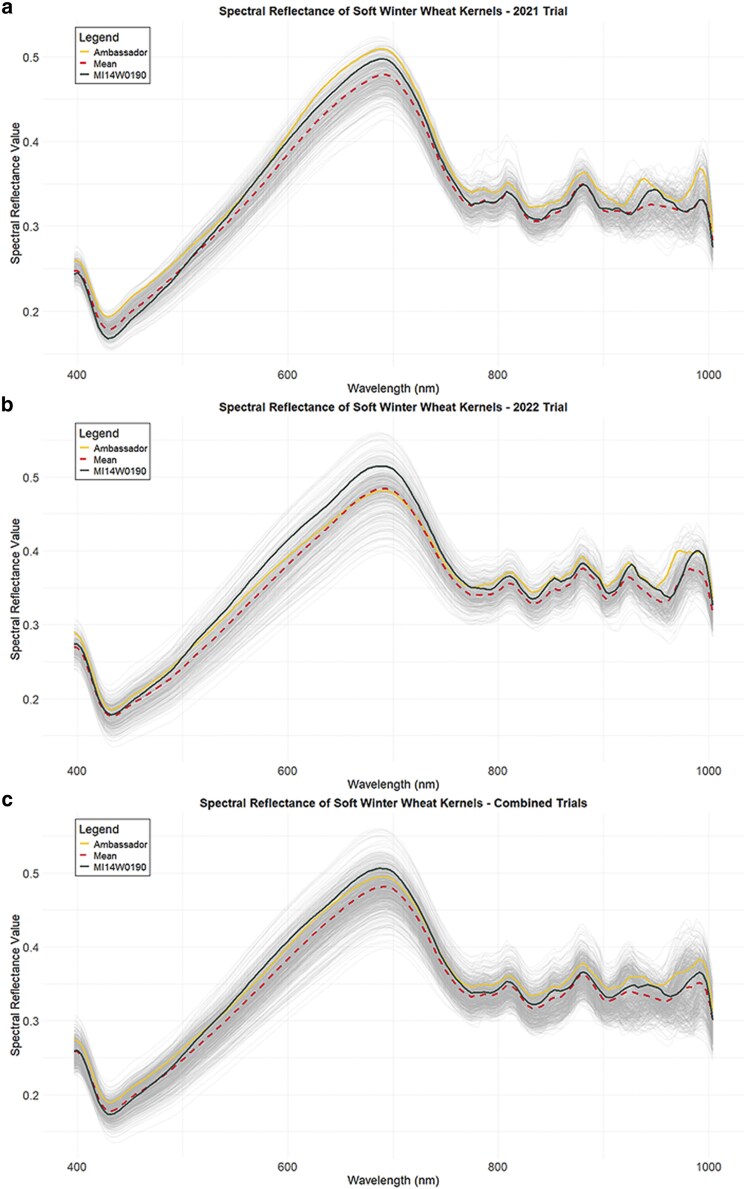
Spectral reflectance profile at the 400 to 1,000 nm range of genotyped evaluated in (a) 2021 and (b) 2022, as well as in a (c) combined trials. The spectral profile of the FHB-resistant check MI14W0190 is indicated by the blue line and the FHB-susceptible check Ambassador is indicated by the yellow line. The red broken line represents the average spectral profile for all the genotypes tested.

**Fig. 3.**
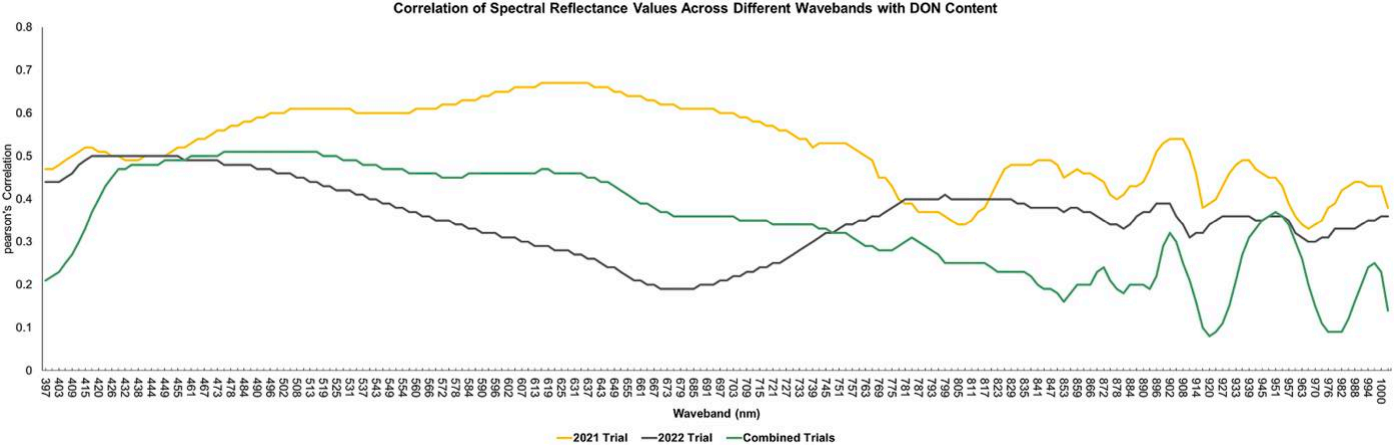
Pearson's correlation of spectral reflectance values with DON for 2021, 2022, and combined 2021 and 2022 trials.

Significant correlations were identified between the 204 wavebands (Fig. 2) in the visible and near-infrared regions and DON content for 2021, 2022, and across years (Fig. 3). In 2021, all VIS wavebands from 409 to 763 nm, along with wavebands from 896 to 911 nm in the NIR region, demonstrated correlations of at least 0.5 with DON content. Wavebands from 496 to 703 nm all demonstrated correlations of at least 0.6 (Figure 3). In 2022, correlations were lower, with only 14 wavebands from 418 to 455 nm reaching 0.5, and visible spectrum correlations ranged from 0.19 to 0.5. Interestingly, several near-infrared wavebands from 800 to 1,000 nm had higher correlations with DON than those in the visible spectrum (Fig. 3). Across years, correlations ranged from 0.21 to 0.51 in the visible spectrum from 400 to 800 nm and 0.09 to 0.37 in the near-infrared region. A moderate correlation of at least 0.5 was observed in wavebands from 461 to 525 nm (Fig. 3).

### Model comparison for DON prediction: predictive ability

To evaluate and compare DON prediction models trained using phenomic or genomic data, a 5-fold cross-validation with an 80/20 training/testing split was performed 100 times for each model. Using the 2021 trial as the training set, wavebands in the VIS spectrum (Phenomic_VIS) outperformed genomic prediction and phenomic prediction using all wavebands (Phenomic_VIS/NIR) across models. Bayes B, Bayes C, and RRBLUP achieved the highest predictive ability at 0.82, followed by Bayes A and Bayesian LASSO (Table 1). In the 2022 trial, predictive ability was lower across all models and phenomic prediction using wavebands in the VIS spectrum again were better predictors of DON. RRBLUP had the highest had the highest predictive ability of 0.70 in 2022, followed by Bayes B and Bayes A (Table 1).

**Table 1.** Cross validation predictive abilities (Pearson's correlations) of phenomic and genomic prediction models in 2021, 2022, and combined trials.

Model	Predictor	2021	2022	2021 and 2022 combined
Bayes A	Geno	0.69 ± 0.058	0.43 ± 0.096	0.61 ± 0.063
	Pheno_V	0.81 ± 0.047	0.65 ± 0.07	0.69 ± 0.049
	Pheno_V/N	0.77 ± 0.057	0.59 ± 0.078	0.68 ± 0.057
Bayes B	Geno	0.70 ± 0.065	0.45 ± 0.09	0.61 ± 0.060
	Pheno_V	0.82 ± 0.045	0.67 ± 0.082	0.68 ± 0.063
	Pheno_V/N	0.81 ± 0.046	0.63 ± 0.07	0.69 ± 0.056
Bayes C	Geno	0.70 ± 0.062	0.42 ± 0.11	0.62 ± 0.061
	Pheno_V	0.82 ± 0.042	0.63 ± 0.075	0.71 ± 0.052
	Pheno_V/N	0.81 ± 0.054	0.59 ± 0.092	0.68 ± 0.061
Bayesian LASSO	Geno	0.71 ± 0.059	0.43 ± 0.102	0.61 ± 0.065
	Pheno_V	0.81 ± 0.05	0.65 ± 0.075	0.69 ± 0.050
	Pheno_V/N	0.71 ± 0.068	0.58 ± 0.09	0.64 ± 0.060
BRR	Geno	0.70 ± 0.061	0.43 ± 0.088	0.61 ± 0.070
	Pheno_V	0.81 ± 0.051	0.61 ± 0.076	0.69 ± 0.055
	Pheno_V/N	0.67 ± 0.057	0.57 ± 0.096	0.60 ± 0.060
Random Forest	Geno	0.71 ± 0.061	0.39 ± 0.087	0.61 ± 0.056
	Pheno_V	0.68 ± 0.062	0.57 ± 0.07	0.66 ± 0.051
	Pheno_V/N	0.69 ± 0.043	0.55 ± 0.075	0.65 ± 0.049
RRBLUP	Geno	0.41 ± 0.14	0.68 ± 0.076	0.67 ± 0.062
	Pheno_V	0.82 ± 0.04	0.70 ± 0.069	0.70 ± 0.050
	Pheno_V/N	0.79 ± 0.044	0.63 ± 0.064	0.69 ± 0.049
XGBoost	Geno	0.61 ± 0.088	0.33 ± 0.096	0.60 ± 0.076
	Pheno_V	0.64 ± 0.089	0.53 ± 0.095	0.64 ± 0.047
	Pheno_V/N	0.62 ± 0.095	0.49 ± 0.08	0.61 ± 0.062

Bayesian LASSO, Bayesian Least Absolute Shrinkage and Selection Operator; BRR, Bayesian Ridge Regression; RRBLUP, Ridge Regression Best Linear Unbiased Prediction; XGBoost, Extreme Gradient Boosting; VIS, Visible Light Spectrum; VIS/NIR, Visible and Near Infrared Light Spectrum; Geno, Genomic prediction; Pheno_V, Phenomic prediction using wavebands in the VIS spectrum; Pheno_V/N, Phenomic Prediction using wavebands in the VIS and NIR light spectrums.

To develop a multiyear prediction, BLUEs were generated from genotypes tested in 2021 and 2022. Multiyear phenomic predictions consistently outperformed genomic predictions across all models (Table 1). As with individual trials as the training set, wavebands in the VIS spectrum had significantly higher predictive ability, with Bayes C achieving the highest at 0.71.

### Model comparison for DON prediction: genetic correlation

Genetic correlation was also assessed to further compare phenomic and genomic prediction models from the cross-validations. In the 2021 trial as the training set, higher genetic correlations were observed in predictions using wavebands in the VIS spectrum (Pheno_VIS) (Table 2). RRBLUP showed the highest genetic correlation (0.83), whereas Bayesian models showed slightly lower and similar genetic correlations (0.82) (Table 2). Using the 2022 trial, similar observations were obtained, where predictions using wavebands in the VIS spectrum (Pheno_VIS) showed higher genetic correlations. Bayes B and RRBLUP recorded the highest genetic correlations (0.67) followed by Bayes A and Bayesian LASSO (Table 2).

**Table 2.** Genetic correlations from cross-validation of phenomic and genomic prediction models in 2021, 2022, and combined trials.

Model	Predictor	2021	2022	2021 and 2022 combined
Bayes A	Geno	0.72 ± 0.068	0.56 ± 0.114	0.69 ± 0.062
	Pheno_V	0.82 ± 0.040	0.66 ± 0.097	0.72 ± 0.055
	Pheno_V/N	0.81 ± 0.047	0.63 ± 0.090	0.71 ± 0.060
Bayes B	Geno	0.73 ± 0.057	0.54 ± 0.126	0.68 ± 0.055
	Pheno_V	0.82 ± 0.044	0.67 ± 0.091	0.71 ± 0.053
	Pheno_V/N	0.81 ± 0.048	0.64 ± 0.093	0.71 ± 0.055
Bayes C	Geno	0.74 ± 0.057	0.54 ± 0.126	0.69 ± 0.059
	Pheno_V	0.82 ± 0.044	0.64 ± 0.101	0.70 ± 0.060
	Pheno_V/N	0.81 ± 0.050	0.59 ± 0.091	0.70 ± 0.052
Bayesian LASSO	Geno	0.72 ± 0.057	0.53 ± 0.116	0.68 ± 0.064
	Pheno_V	0.82 ± 0.041	0.66 ± 0.096	0.72 ± 0.046
	Pheno_V/N	0.79 ± 0.046	0.58 ± 0.093	0.71 ± 0.055
BRR	Geno	0.72 ± 0.069	0.52 ± 0.133	0.68 ± 0.064
	Pheno_V	0.82 ± 0.041	0.65 ± 0.087	0.72 ± 0.044
	Pheno_V/N	0.81 ± 0.048	0.58 ± 0.104	0.70 ± 0.059
Random Forest	Geno	0.74 ± 0.060	0.54 ± 0.101	0.71 ± 0.061
	Pheno_V	0.71 ± 0.059	0.54 ± 0.098	0.67 ± 0.048
	Pheno_V/N	0.70 ± 0.062	0.54 ± 0.089	0.67 ± 0.053
RRBLUP	Geno	0.45 ± 0.158	0.73 ± 0.068	0.71 ± 0.062
	Pheno_V	0.83 ± 0.041	0.67 ± 0.080	0.72 ± 0.049
	Pheno_V/N	0.80 ± 0.045	0.61 ± 0.076	0.71 ± 0.048
XGBoost	Geno	0.72 ± 0.053	0.47 ± 0.118	0.69 ± 0.065
	Pheno_V	0.66 ± 0.067	0.52 ± 0.099	0.64 ± 0.056
	Pheno_V/N	0.66 ± 0.070	0.49 ± 0.092	0.62 ± 0.054

Bayesian LASSO, Bayesian Least Absolute Shrinkage and Selection Operator; BRR, Bayesian Ridge Regression; RRBLUP, Ridge Regression Best Linear Unbiased Prediction; XGBoost, Extreme Gradient Boosting; VIS, Visible Light Spectrum; VIS/NIR, Visible and Near Infrared Light Spectrum; Geno, Genomic prediction; Pheno_V, Phenomic prediction using wavebands in the VIS spectrum; Pheno_V/N, Phenomic Prediction using wavebands in the VIS and NIR light spectrums.

Multiyear phenomic predictions outperformed genomic predictions, except in Random Forest and XGBoost where genetic correlations from genomic predictions were higher. The highest genetic correlations were recorded using Bayes A, Bayesian LASSO, and RRBLUP (Table 2).

### Model comparison for DON prediction: prediction accuracy

Prediction accuracy, the product between genetic correlations and square root of the estimated heritability of the actual DON content in the testing set, was also determined to fully assess the potential utility of the trained models for DON prediction. In the 2021 trial, phenomic predictions using wavebands in the VIS spectrum (Pheno_VIS) showed higher prediction accuracy over using the full waveband ranges or with genomic predictions (0.60 to 0.77) (Table 3). However, in Random Forest and XGBoost, higher prediction accuracy was observed in genomic predictions over phenomic predictions (Table 3). In the 2022 trial, phenomic predictions using the wavebands in the VIS spectrum were higher than genomic prediction (0.26 to 0.33), with Bayes C where prediction accuracy being the highest using the full waveband ranges (0.49) (Table 3). In addition, genomic prediction using RRBLUP resulted in the highest prediction accuracy at 0.50 (Table 3).

**Table 3.** Prediction accuracy from cross-validation of phenomic and genomic prediction models in 2021, 2022, and combined trials.

Model	Predictor	2021	2022	2021 and 2022 combined
Bayes A	Geno	0.67 ± 0.010	0.22 ± 0.146	0.51 ± 0.101
	Pheno_V	0.76 ± 0.099	0.33 ± 0.201	0.55 ± 0.105
	Pheno_V/N	0.73 ± 0.111	0.31 ± 0.196	0.53 ± 0.120
Bayes B	Geno	0.65 ± 0.102	0.26 ± 0.159	0.53 ± 0.103
	Pheno_V	0.76 ± 0.103	0.30 ± 0.189	0.54 ± 0.125
	Pheno_V/N	0.74 ± 0.118	0.30 ± 0.198	0.53 ± 0.117
Bayes C	Geno	0.65 ± 0.100	0.24 ± 0.161	0.53 ± 0.095
	Pheno_V	0.74 ± 0.108	0.31 ± 0.188	0.52 ± 0.138
	Pheno_V/N	0.74 ± 0.108	0.49 ± 0.166	0.51 ± 0.104
Bayesian LASSO	Geno	0.66 ± 0.086	0.25 ± 0.158	0.53 ± 0.095
	Pheno_V	0.77 ± 0.091	0.32 ± 0.188	0.57 ± 0.105
	Pheno_V/N	0.73 ± 0.087	0.29 ± 0.161	0.54 ± 0.107
BRR	Geno	0.65 ± 0.101	0.22 ± 0.152	0.53 ± 0.094
	Pheno_V	0.77 ± 0.094	0.31 ± 0.195	0.55 ± 0.105
	Pheno_V/N	0.74 ± 0.097	0.27 ± 0.178	0.54 ± 0.110
Random Forest	Geno	0.68 ± 0.082	0.25 ± 0.120	0.52 ± 0.079
	Pheno_V	0.65 ± 0.082	0.27 ± 0.149	0.50 ± 0.089
	Pheno_V/N	0.65 ± 0.075	0.26 ± 0.136	0.50 ± 0.093
RRBLUP	Geno	0.32 ± 0.176	0.50 ± 0.176	0.51 ± 0.139
	Pheno_V	0.76 ± 0.083	0.31 ± 0.167	0.54 ± 0.095
	Pheno_V/N	0.74 ± 0.082	0.28 ± 0.149	0.53 ± 0.093
XGBoost	Geno	0.66 ± 0.079	0.22 ± 0.107	0.51 ± 0.070
	Pheno_V	0.60 ± 0.088	0.26 ± 0.146	0.49 ± 0.087
	Pheno_V/N	0.60 ± 0.088	0.24 ± 0.129	0.48 ± 0.087

Bayesian LASSO, Bayesian Least Absolute Shrinkage and Selection Operator; BRR, Bayesian Ridge Regression; RRBLUP, Ridge Regression Best Linear Unbiased Prediction; XGBoost, Extreme Gradient Boosting; VIS, Visible Light Spectrum; VIS/NIR, Visible and Near Infrared Light Spectrum; Geno, Genomic prediction; Pheno_V, Phenomic prediction using wavebands in the VIS spectrum; Pheno_V/N, Phenomic Prediction using wavebands in the VIS and NIR light spectrums.

In the multiyear predictions, phenomic and genomic prediction accuracy appears to be somewhat similar, with all of the prediction accuracies, regardless of whether it is phenomic or genomic prediction, ranged from 0.48 to 0.57 (Table 3).

### Feature selection

**Fig. 4.**
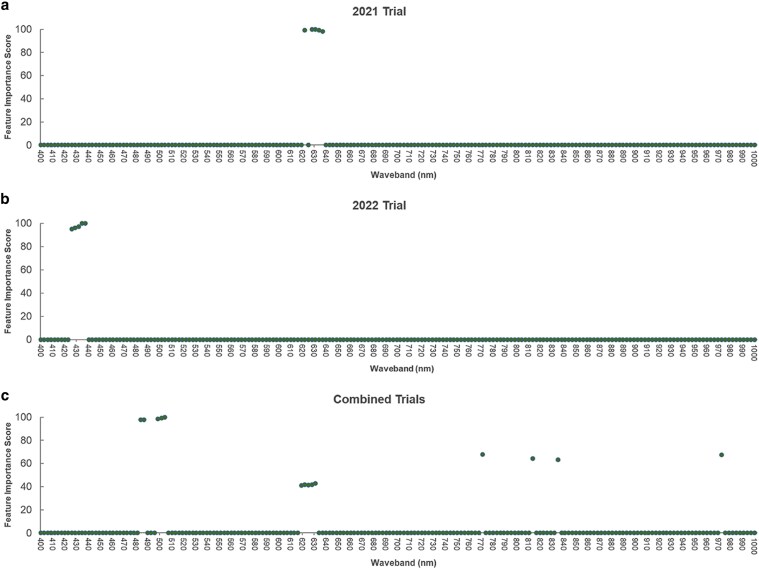
Feature selection based on recursive partitioning and regressive tree models. Feature importance scores are presented for the wavebands in the VIS and NIR regions from the (a) 2021 trial, (b) 2022 trial, and (c) 2021 and 2022 trials combined.

Wavebands in the VIS spectrum from 400 to 800 nm were identified to have the most important wavebands for both individual years and across years using recursive partitioning and regressive tree model (Fig. 4). Specifically, in the 2021 trial, wavebands at 622, 628, 631, 634, and 637 nm, all located in the red light spectrum, were identified as the most informative wavebands having feature importance score of 98 to 100. Interestingly in the 2022 trial, wavebands in the blue light spectrum—426, 429, 431, 435, and 438 nm—were identified to be the most informative wavebands with feature importance scores ranging from 95 to 100. In the combined trials, most informative wavebands having feature importance scores of 40 to 100 were identified across the entire spectrum, including 4 wavebands at the NIR region—814, 835, 972, and 1,004 nm. Majority of the informative wavebands still are located in the VIS spectrum—484, 487, 499, 502, 505, 619, 622, 625, 628, 631, and 772 nm. Overall, wavebands in the red and green light spectrum appear have the most important wavebands associated with DON concentration in infected wheat kernels.

### Predictive ability of trained models in F_4:5_ selection candidates

After evaluating phenomic and genomic model performance within training sets, we evaluated the model predictive ability outside of the training sets on F_4:5_ selection candidates evaluated in 2022 and 2023. All models were used in forward prediction and model blending.

When using the 2021 training set to predict DON content in the 2022 selection candidates, blended phenomic and genomic predictions demonstrated higher phenotypic correlations with observed DON content than the blended genomic predictions and the blended phenomic predictions, with the latter having slightly higher phenotypic correlation with DON. A similar observation was also obtained when the 2021 training set was used to predict the 2023 selection candidates (Table 4).

**Table 4.** Phenotypic correlation, prediction accuracy, and indirect selection accuracy of blended phenomic, genomic, and combined phenomic predictions in the selection candidates predicted using different training sets.

Training set	Selection candidate	rP			rG			$H_{I}^{2}$			Acc_(I)_			Acc_(A)_		
		Ph	G	Ph + G	Ph	G	Ph + G	Ph	G	Ph + G	Ph	G	Ph + G	Ph	G	Ph + G
2021	2022 F_4:5_	0.12	0.15	0.18	0.02	0.28	0.26	0.42	0.34	0.45	0.015	0.16	0.14	0.01	0.12	0.11
	2023 F_4:5_	0.19	0.21	0.22	0.19	0.43	0.35	0.04	0.03	0.05	0.04	0.08	0.08	0.05	0.12	0.10
2022	2022 F_4:5_	0.49	0.27	0.47	0.33	0.50	0.58	0.51	0.51	0.60	0.24	0.36	0.45	0.14	0.21	0.25
	2023 F_4:5_	0.52	0.14	0.41	0.47	0.33	0.50	0.13	0.11	0.20	0.17	0.11	0.22	0.13	0.09	0.14
2021 + 2022	2022 F_4:5_	0.30	0.20	0.31	0.14	0.36	0.38	0.44	0.37	0.50	0.09	0.22	0.27	0.06	0.16	0.16
	2023 F_4:5_	0.38	0.18	0.31	0.31	0.38	0.41	0.07	0.04	0.08	0.08	0.08	0.12	0.07	0.11	0.12

Ph, blended phenomic gredictions; G, blended genomic predictions; Ph + G, blended phenomic and genomic predictions; r_P_, phenotypic correlation of the blended predictions with observed DON content in the selection candidates; r_G_, genetic correlation of the blended predictions with observed DON content in the selection candidates; $H_{I}^{2}$, estimated broad-sense heritability calculated from the blended predicted DON values; Acc_(I)_, indirect selection accuracy; Acc_(A)_, prediction accuracy.

Genotypes from the 2022 trial were used to predict DON content in selection candidates also evaluated in 2022. The blended phenomic predictions had a higher phenotypic correlation with observed DON content than the blended genomic predictions and blended phenomic and genomic predictions, with the latter having relatively similar phenotypic correlation with blended phenomic predictions (Table 4). A similar pattern was observed in predicting the 2023 selection candidates with 2022 trial as the training set; however, the difference between the blended phenomic predictions compared to blended phenomic and genomic predictions were more profound (Table 4).

When using BLUEs across years for training to predict DON content in the 2022 selection candidates, the blended phenomic and genomic predictions demonstrated a slightly higher phenotypic correlation with observed DON content, compared to blended genomic predictions and blended phenomic predictions (Table 4). Using BLUEs across years to predict DON content in 2023 selection candidates, the blended phenomic predictions had higher phenotypic correlation with observed DON content than blended phenomic and genomic predictions. Both outperformed the blended genomic predictions (Table 4).

### Genetic correlation of predicted and observed DON content in the selection candidates

We evaluated the genetic correlation between predicted DON values and observed DON content in the selection candidates. Genomic EBVs (GEBVs) generated from genomic predictions are primarily based on additive genetic effects and assume no environmental influence (Meuwissen et al. 2001; Habier et al. 2010). In contrast, phenomic predictions incorporate nonadditive effects and can include genotype-by-environment interactions and typically predict phenotype rather than genetic merit (Rincent et al. 2018).

The blended phenomic and genomic predictions demonstrated higher genetic correlation with observed DON content (Table 4) in 2022 F_4:5_ selection candidates predicted using the 2022 trial and combined trials as the training set compared to blended phenomic or blended genomic predictions. A similar observation was also obtained in the 2023 selection candidates (Table 4). For both 2022 and 2023 selection candidates, when 2021 trial was used as the training set, a higher genetic correlation was observed in blended genomic predictions compared to either blended phenomic or blended genomic and phenomic predictions (Table 4). Overall, blended phenomic and genomic predictions appear to result in higher genetic correlations over either blended genomic or blended phenomic predictions.

### Prediction accuracy and indirect selection accuracy estimation from forward prediction of DON content in the selection candidates

For the 2022 selection candidates, a higher prediction accuracy was obtained in the blended phenomic and genomic predictions and in blended genomic predictions, with the latter having slightly higher prediction accuracy, when the 2021 trial was used as the training set (Table 4). Using the 2022 trial as the training set, higher prediction accuracy was obtained in the blended phenomic and genomic predictions, whereas similar prediction accuracies were obtained in blended phenomic and genomic predictions and in blended genomic predictions using the combined trials as the training set (Table 4).

For the 2023 selection candidates, a higher prediction accuracy was obtained in the genomic predictions, when the 2021 trial was used as the training set (Table 4). When the 2022 trial and combined trials were used as the training set, the blended phenomic and genomic predictions resulted in higher prediction accuracy (Table 4). Overall, blended phenomic and genomic predictions resulted in higher prediction accuracy, especially over blended phenomic predictions.

Indirect selection accuracy was derived by multiplying the square root of the estimated broad-sense heritability of predicted DON content with the genetic correlation between predicted and observed DON content in selection candidates. For the 2022 selection candidates, higher indirect selection accuracy was observed in blended genomic predictions with 2021 trial as the training set, whereas higher indirect selection accuracy was observed in the blended phenomic and genomic predictions when the 2022 trial and the combined trials were used as the training set (Table 4).

For the 2023 selection candidates, relatively similar indirect selection accuracy was obtained in the blended genomic predictions and in the blended phenomic and genomic predictions using 2021 trial as the training set (Table 4). Higher indirect selection accuracy was obtained, however, in the blended phenomic and genomic predictions (Table 4). Overall, blended phenomic and genomic predictions resulted in higher indirect selection accuracy over blended phenomic and blended genomic predictions. This is likely due to the estimated heritability of predictions from blended phenomic and genomic predictions being relatively higher compared to blended phenomic or blended genomic predictions (Table 4).

### Unsupervised K-means clustering of selection candidates using model blending

Truncation selection can be applied to predicted DON content generated from a single phenomic or genomic prediction model while lending of predictions from multiple models can enable classification of selection candidates for selection. We used unsupervised K-means clustering to separate the selection candidates into groups based on the blending of predictions generated across all models evaluated and used. Two groups were used for clustering based on the results of the Silhouette method.

**Fig. 5.**
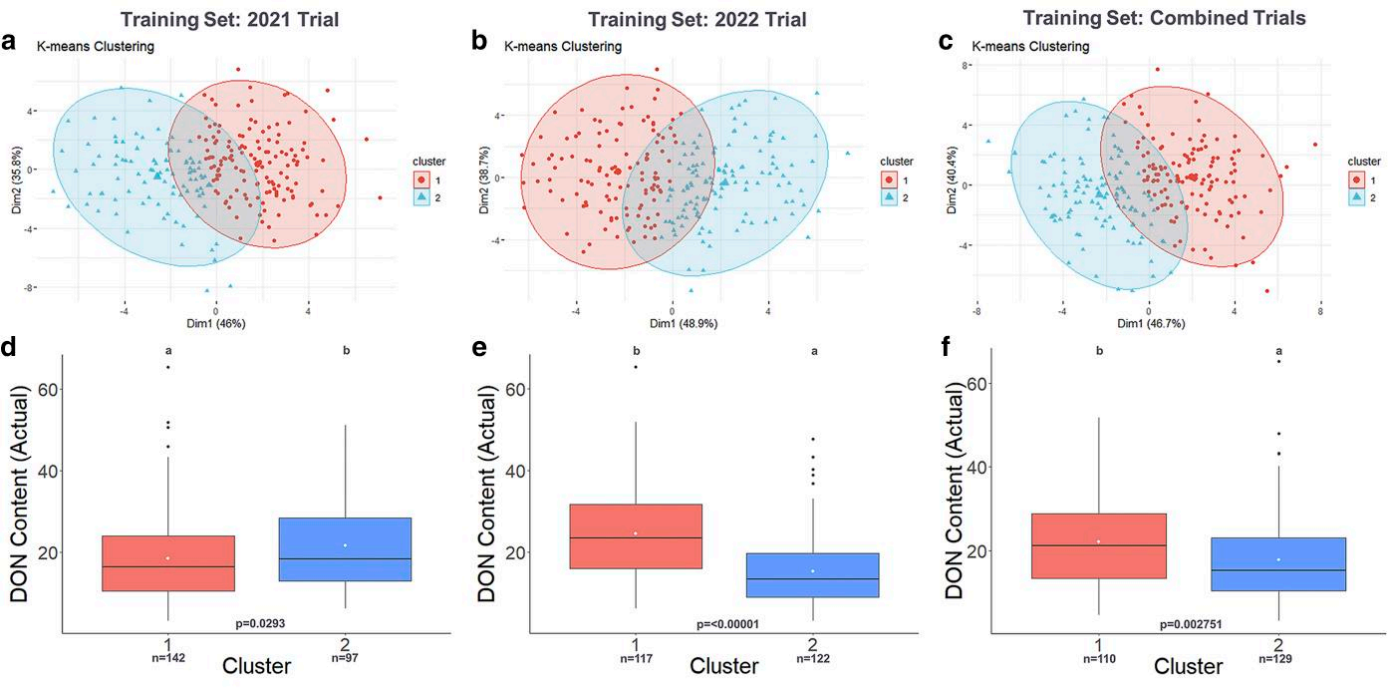
Unsupervised K-means clustering based on blended phenomic and genomic of DON content predictions in 2022 F_4:5_ selection candidates using different training sets. (a to c) Clustering of 2022 F_4:5_ selection candidates using genomically and predicted values. (d to f) Corresponding boxplots showing the actual DON content of the genotypes in clusters 1 and 2 grouped based on phenomically and genomically predicted values. The white dot represents the mean actual DON content of genotypes belonging to clusters 1 and 2. Means with the same letter are not significantly different at alpha 0.05.

Clustering of the 2022 F_4:5_ selection candidates with combined phenomic and genomic predictions generated 2 distinct groups, Low DON and High DON, based on differences in the mean DON level of each group. These clusters demonstrated significantly different mean DON content using training sets from 2021 (*P* = 0.02294), 2022 (*P* = 1.074 × 10^−11^), and the combined 2021 and 2022 trials (*P* = 0.002751) (Fig. 5, Supplementary Tables 5 to 7). Significant differences between Low and High DON groups were also observed from both blended phenomic and blended genomic predictions using the 2022 trial and the combined 2021 and 2022 trials as the training sets (Supplementary Figs. 1 and 2, Supplementary Tables 5 and 7). However, when the 2021 trial was used as the training set to predict 2022 selection candidates using only the blended genomic or blended phenomic predictions, no significant difference was observed between Low and High DON (Supplementary Fig. 1 and Supplementary Tables 2 and 5) groups.

**Fig. 6.**
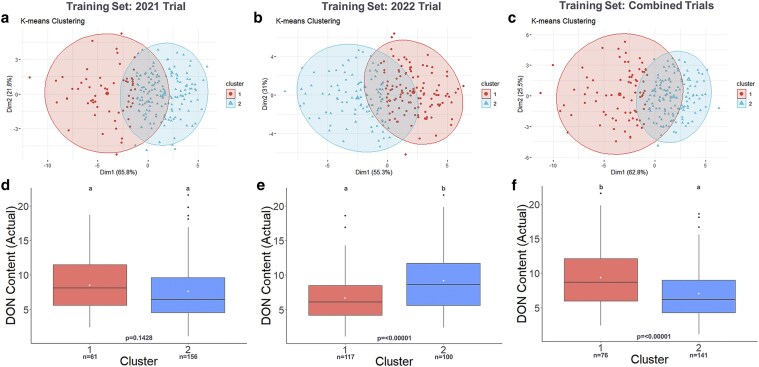
Unsupervised K-means clustering based on blended phenomic and genomic of DON content predictions in 2022 F_4:5_ selection candidates using different training sets. (a to c) Clustering of 2022 F_4:5_ selection candidates using genomically and predicted values. (d to f) Corresponding boxplots showing the actual DON content of the genotypes in clusters 1 and 2 grouped based on phenomically and genomically predicted values. The white dot represents the mean actual DON content of genotypes belonging to clusters 1 and 2. Means with the same letter are not significantly different at alpha 0.05.

Clustering of the 2023 F_4:5_ selection candidates with combined phenomic and genomic predictions also generated 2 distinct groups with significantly different mean DON content using training sets from 2022 (*P* = 3.604 × 10^−6^) and combined 2021 and 2022 trials (*P* = 3.605 × 10^−5^) (Fig. 6, Supplementary Tables 9 and 10). Combined phenomic and genomic predictions using the 2021 trial as the training set yielded no significant difference between groups of 2023 selection candidates (Fig. 6, Supplementary Table 8). However, significant differences between groups of 2023 selection candidates were generated from the blended phenomic predictions using the 2021 trial (*P* = 0.007936), 2022 trial (*P* = 4.018 × 10^−12^), and combined 2021 and 2022 trials (*P* = 1.536 × 10^−5^) as the training sets (Supplementary Fig. 3, Supplementary Tables 9 and 10). Significant differences between groups were observed using the blended genomic predictions with the 2021 trial as the training set (*P* = 0.02376) (Supplementary Fig. 4, Supplementary Table 8).

## Discussion

### Hyperspectral imaging of DON-infected kernels and phenomic prediction

Hyperspectral imaging offers a rapid, cost-effective, and nondestructive approach to evaluate DON content in wheat kernels in comparison to labor and resource intensive chemometric approaches (Femenias et al. 2020). Hyperspectral imaging methods produce multidimensional data from hundreds of wavebands and identifying the key wavebands provides crucial information on the basis of observed variation in DON content. In our study, the most important wavebands were found in the VIS spectrum, particularly in the red light range around 600 nm. Noise was observed in the reflectance values beyond 750 nm even after smoothing using the Savitzky–Golay approach possibly due to inaccurate collection of spectral data in this region. In this study, LED was used as the light source, potentially affecting the wavelengths of light reflected in the NIR region, as most LEDs do not emit light beyond the 800 nm range. This may have also led to the relatively lower correlation of the wavebands in the NIR region with DON compared to wavebands in the VIS region. Therefore, using light sources emitting or covering the full VIS/NIR range would be more advantageous to better estimate the relationship between wavebands in the NIR region and DON. Notable differences were found between the wavebands identified as important in 2021 and 2022 trials. The correlations between individual wavebands and DON content also differed between years. This variation could be due to the environmental factors peculiar to each year or differences in infection levels across the disease nursery, which could introduce error into DON observations for individual genotypes.

Despite noise in the NIR region and the differences between years, the importance of the VIS spectrum between 410 and 640 nm is in agreement with previous reports of wavebands associated with *Fusarium*-damaged kernels (Shahin and Symons 2011, 2012; Ropelewska and Zapotoczny 2018). Cross-validations done in this study highlight the observation that wavebands in the VIS spectrum are sufficient to predict DON content compared to using the entire spectrum of VIS and NIR light.

### Comparison of phenomic and genomic predictions for DON

Genomic prediction employs genetic information, usually SNPs, which capture additive effects primarily from small effect quantitative trait loci (Cerrudo et al. 2018). Since genetic information is stable across generations and is essentially unaffected by the environment, genomic prediction enables the estimation of a candidate's genetic breeding value or genetic merit (Meuwissen et al. 2001). This estimation is essential for ranking and identifying candidates with superior performance and for selecting promising parents for population development. Several studies have demonstrated the use of genomic prediction for low DON content (Rutkoski et al. 2012; Sallam et al. 2015; Gaire et al. 2022). The main advantage of genomic prediction is the ability to estimate breeding values of selection candidates early in the breeding cycle. However, genomic prediction can be costly due to the required genotyping, and environmental effects on the trait of interest must still be considered.

Phenomic predictions capture phenotypic variation that reflects both genetic and environmental factors and account for nonadditive effects (Rincent et al. 2018; Robert et al. 2022a, 2022b). Therefore, phenomic prediction focuses on predicting the phenotype rather than solely the genetic merit or breeding value making phenomic prediction an excellent approach for selection in complex traits (Zhu et al. 2021), as in the case of DON content. Several works have laid the foundation for use of hyperspectral imaging for DON prediction (Femenias et al. 2020 and references therein). Among these, Alisaac et al. (2019) demonstrated the use of hyperspectral imaging in assessing mycotoxin, including DON, in wheat kernels. However, unlike in our study, the wavebands generated were not used as predictors for predicting DON content, rather, direct comparisons of spectral signatures in relation to DON content were made. Su et al. (2020) did use the spectral reflectance values for DON prediction using locally weighted partial least squares regression with relative success in barley kernels.

In this study, phenomic prediction demonstrated high predictive ability consistent with other studies across different crop species (Parmley et al. 2019; Galan et al. 2020; Lane et al. 2020; Brault et al. 2022), including wheat (Cuevas et al. 2019; Krause et al. 2019; Liu et al. 2024). Bayesian generalized linear regression models (Bayes A, Bayes B, Bayes C, Bayesian LASSO, and Bayesian Ridge Regression) demonstrated relatively higher predictive ability in cross-validation studies compared to the machine learning models Random Forest and XGBoost. Furthermore, our results agree with the observations made by Zhu et al. (2021) where phenomic predictive ability using RRBLUP, Bayes B, and Bayes C was higher than random forest and gradient boosting approaches. This suggests a linear relationship between the phenomic signals captured by hyperspectral imaging and DON content in wheat kernels.

Recently, however, Wang et al. (2023) argued that the comparison between genomic and phenomic predictions simply by assessing the phenotypic correlation (predictive ability), especially from cross-validation schemes, could be misleading. As genomic prediction estimates the breeding value or genetic merit of the target trait, in our case DON, Wang et al. (2023) proposed the comparison of between genomic and phenomic predictions should be assessed using prediction accuracy, which takes into account the genetic correlation between the predicted and the actual trait value, and the heritability of the target trait, rather than solely relying on Pearson's correlation between observed and predicted phenotypic values. We agree that using predictive ability, solely, in comparing GEBVs with predicted phenotype may be misleading. Predicted phenotypes would be more correlated with the actual trait value than GEBVs, especially when the predictors used (e.g. phenomic data) have some level of correlation with the target trait, which will inflate the observed Pearson's correlation between the predicted and actual phenotypes. In our case, higher genetic correlations were still observed in phenomic predictions compared to genomic predictions. More importantly, overall, we observe a higher prediction accuracy in phenomic prediction compared to genomic predictions, especially in the 2021 and 2022 trials when independently used as the training set. To our knowledge, this is the first work using prediction accuracy and genetic correlation as metrics comparing genomic predictions of DON mycotoxin with phenomic predictions based on hyperspectral imaging.

### Training set composition influences forward prediction in selection candidates

The choice of the training set significantly impacted predictions of DON content in both phenomic and genomic predictions. While phenomic prediction generally demonstrated higher predictive ability, genetic correlation, and prediction accuracy than genomic prediction, the values observed varied depending on the training set used.

The models trained with phenomic data from the 2022 trial demonstrated relatively higher predictive ability in the F_4:5_ selection candidates compared to models trained with 2021 data and the combined 2021 and 2022 BLUEs, especially in the 2022 F_4:5_ selection candidates. This is likely influenced by the training set and selection candidate being in the same environment and receiving relatively similar disease pressure. A similar trend was observed for genomic prediction models. However, there was a larger difference in prediction accuracy between years in genomic prediction models compared to phenomic prediction models.

We also observed variation in the genetic correlations between blended predictions (model averages) and the actual DON content of the selection candidates, depending on the training set used. For the majority of cases, blended genomic predictions demonstrated higher genetic correlations over blended phenomic predictions, except when 2022 trial was used as the training set to predict the 2022 F_4:5_ selection candidates. This further establishes that solely relying on phenotypic correlation, both in cross-validation and forward predictions, may not be sufficient or even misleading.

In addition, the predictive abilities and prediction accuracy observed in cross-validation studies within training sets did not always translate when models were used to predict a set of selection candidates. While cross-validation demonstrated higher predictive abilities and prediction accuracy for phenomic predictions using the 2021 trial, this was not reflected in the prediction of the 2023 selection candidates, where the 2022 training set generated better phenotypic correlations, genetic correlation, and prediction accuracy with observed DON content.

These differences could be driven by the variation among the genotypes included in each training set and their genetic relatedness to the selection candidates (Zhu et al. 2021). Of the 307 genotypes evaluated in 2021, only 37 were evaluated in 2022, due to the dynamic nature of active breeding programs. The selection candidates in 2022 and 2023 were derived from cross combinations of different parental genotypes and therefore have genetic backgrounds differing from the training set. Differences in infection levels and the unique environmental parameters within each year may have contributed to differences in predictive ability. Indeed, Robert et al. (2022a, 2022b) highlight that the environments, years in our case, in which the lines were grown, play a crucial role in predictive ability of spectral reflectance data. The strength of correlation between wavebands and DON differed in 2021 and 2022, likely affecting the performance of phenomic prediction models in forward prediction.

Variation in predictive ability, genetic correlations, and prediction accuracy depending on the predictors used (phenomic or genomic information) and training set used (2021 trial, 2022 trial, and combined trials) prompted us to explore whether combining phenomic and genomic information would be more advantageous. Thapa et al. (2024) demonstrated potential for increasing predictive ability for DON content by incorporating both phenomic and genomic information. In this study, we extend predictions beyond cross-validation and implemented forward predictions of DON content in novel unobserved genotypes and environments. Further, we employed a different approach by blending phenomic and genomic predictions.

### Model averaging and complementation of genomic prediction with phenomic predictions

In this study, the models Bayes A, Bayes B, Bayes C, Bayesian LASSO, Bayesian Ridge Regression, Random Forest, RRBLUP, and XGBoost were used to predict DON content in selection candidates. Given the differing assumptions and fitting of these models, a model averaging approach was explored to enhance prediction accuracy. Phenomic predictions estimate the phenotype, which also captures the nonadditive effects, often confounding environmental effects, whereas genomic predictions estimate the breeding value of selection candidates independent of environmental influences. In this study, we opted to average the predicted values from different models and made comparisons between the blended genomic or phenomic predictions and extend blending to incorporate both phenomic and genomic predictions.

In the majority of cases, the genetic correlation between the blended predictions from phenomic models and observed DON in the selection candidates was higher than that from genomic models. Most importantly, the estimated heritability in the predicted values was always higher in the blended phenomic and genomic predictions, compared to blended phenomic or blended genomic predictions. This observation also held true for indirect selection accuracy, suggesting that blending phenomic predictions can effectively predict DON content in wheat. This work demonstrates the advantage and utility of blending phenomic and genomic predictions in forward prediction, through simple model averaging.

Considering these observations, both phenomic and genomic predictions can be used whenever feasible and when resources allow in order to implement a balanced selection approach that leverages the strengths of both methods. However, as phenomic information potentially captures nonadditive and environmental effects (Rincent et al. 2018)), evaluating prediction models that take into account nonadditive effects (Jiang and Reif 2015; Onogi et al. 2021; Kristensen et al. 2023; Chen et al. 2025) including dominance and epistatic effects would provide an even more compelling comparison between phenomic and genomic predictions. Multimodal neural networks (Montesinos-López et al. 2024) and multikernel learning (Briscik et al. 2024) approaches present additional opportunities for the integration phenomic and genomic information. We advise the inclusion of genetic correlations, prediction accuracy described by Wang et al. (2023) and indirect selection accuracy described by Lopez-Cruz et al. (2020) to avoid misleading interpretations, both in cross-validation and forward predictions, rather than solely relying on predictive ability assessment by phenotypic (Pearson's) correlations.

### Clustering-model blending of selection candidates

Unsupervised K-means clustering was explored as a model blending approach to group selection candidates based on their predicted DON contents. Rather than applying truncation selection on predicted values from multiple phenomic or genomic prediction models, the predictions from multiple models were used as variables for clustering. Clustering of selection candidates using blended predictions generated 2 groups that differed significantly for mean observed DON content. This approach shows promise in narrowing the pool of selection candidates, especially when dealing with a large number of potential selection candidates.

### Conclusion

Phenomic prediction of DON content in wheat grains using reflectance values derived from hyperspectral imaging demonstrated overall higher predictive ability and prediction accuracy than genomic prediction. Phenomic predictions resulted in varying phenotypic correlations, genetic correlations, prediction accuracies, and indirect selection accuracies depending on the training sets used for forward prediction. Blending phenomic and genomic prediction models through simple model averaging was found to be a straightforward mechanism to potentially account for both genetic and environmental influences on the trait of interest. Additionally, clustering of selection candidates using blended predictions offers an approach to leverage both phenomic and genomic predictions in making selection decisions. Careful consideration is required for training set design and implementation of phenomic predictions as the inflated predictive ability from cross-validations does not always translate to forward predictions. Additional metrics including genetic correlation, prediction accuracy, and indirect selection accuracy should be assessed to compare models and approaches, especially when comparing phenomic and genomic predictions. This study highlights a mechanism to develop and leverage both phenomic and genomic predictions in selection decisions in an active plant breeding program.

## Supplementary Material

jkaf176_Supplementary_Data

## Data Availability

Hyperspectral reflectance data are publicly available at https://doi.org/10.5061/dryad.d2547d8bx. Genome-wide SNP marker data are publicly available at https://doi.org/10.5061/dryad.d2547d8bx. DON mycotoxin values are available in Supplementary Tables 2, 3, and 4. Supplementary Figs. 1 to 4 contain boxplots showing model average of predicted DON content and actual DON content of selection candidates grouped predicted values by unsupervised k-means clustering. Supplementary Table 1 shows ANOVA conducted for DON content, while Supplementary Tables 2 to 4 show the BLUEs for DON of the genotypes used for model training. Supplementary Tables 5 to 10 show the detailed results of unsupervised k-means clustering conducted in the selection candidates. Supplemental material available at G3 online.
