# Supplementary material for: Genomic and hyperspectral imaging-based prediction blending enables selection for reduced deoxynivalenol content in wheat grains
Source: G3 (Bethesda). 2025 Aug 6;15(10):jkaf176. doi: 10.1093/g3journal/jkaf176 (PMC12506668; doi:10.1093/g3journal/jkaf176)
Supplement: jkaf176_Supplementary_Data [file jkaf176_supplementary_data.zip › Supplemental_Figures_G3-2025-405941.docx]

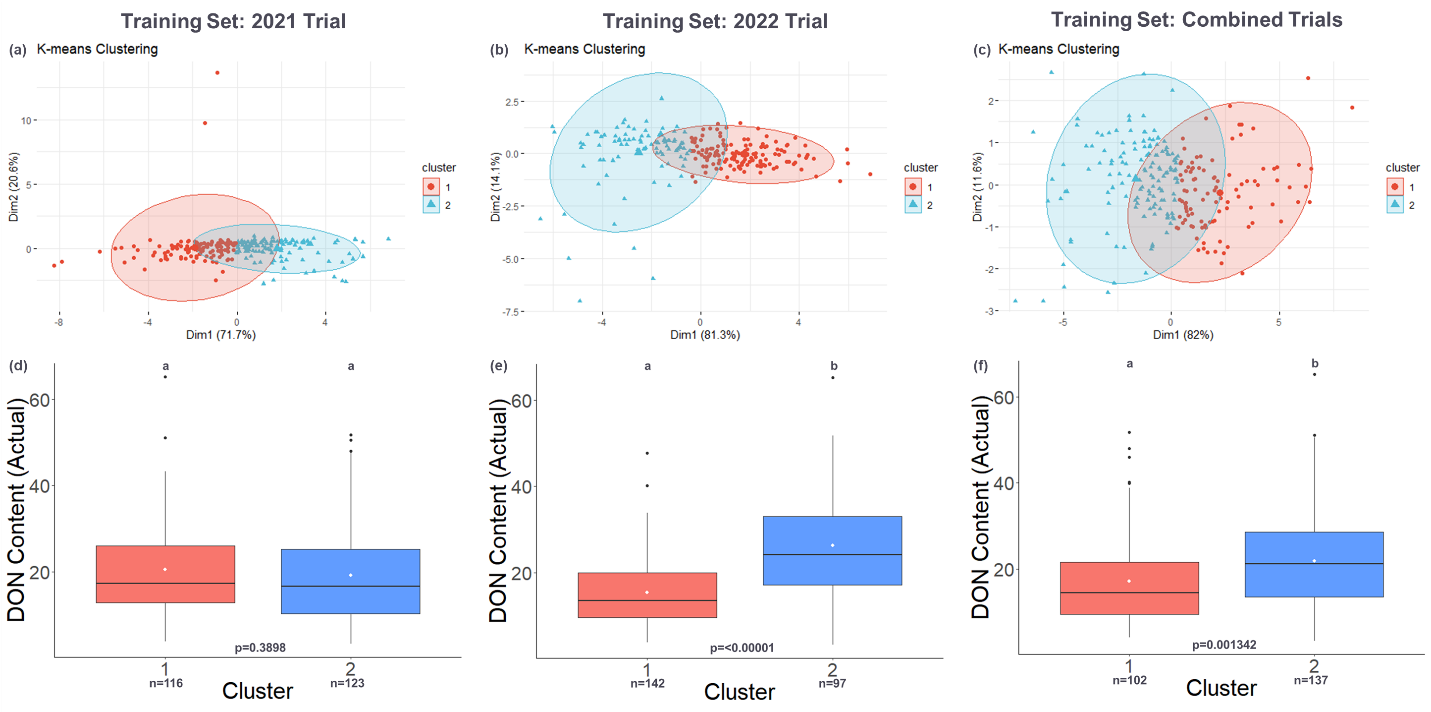


**Supplementary Figure 1.** Unsupervised K-means clustering based on blended phenomic predictions of DON content in 2022 F_4:5_ selection candidates using different training sets. (a, b, c) Clustering of 2022 F_4:5_ selection candidates using genomically and predicted values. (d,e,f) Corresponding boxplots showing the actual DON content of the genotypes in Clusters 1 and 2 grouped based on phenomically and genomically predicted values. White dot represents the mean actual DON content of genotypes belonging to Clusters 1 and 2. Means with the same letter are not significantly different at alpha 0.05


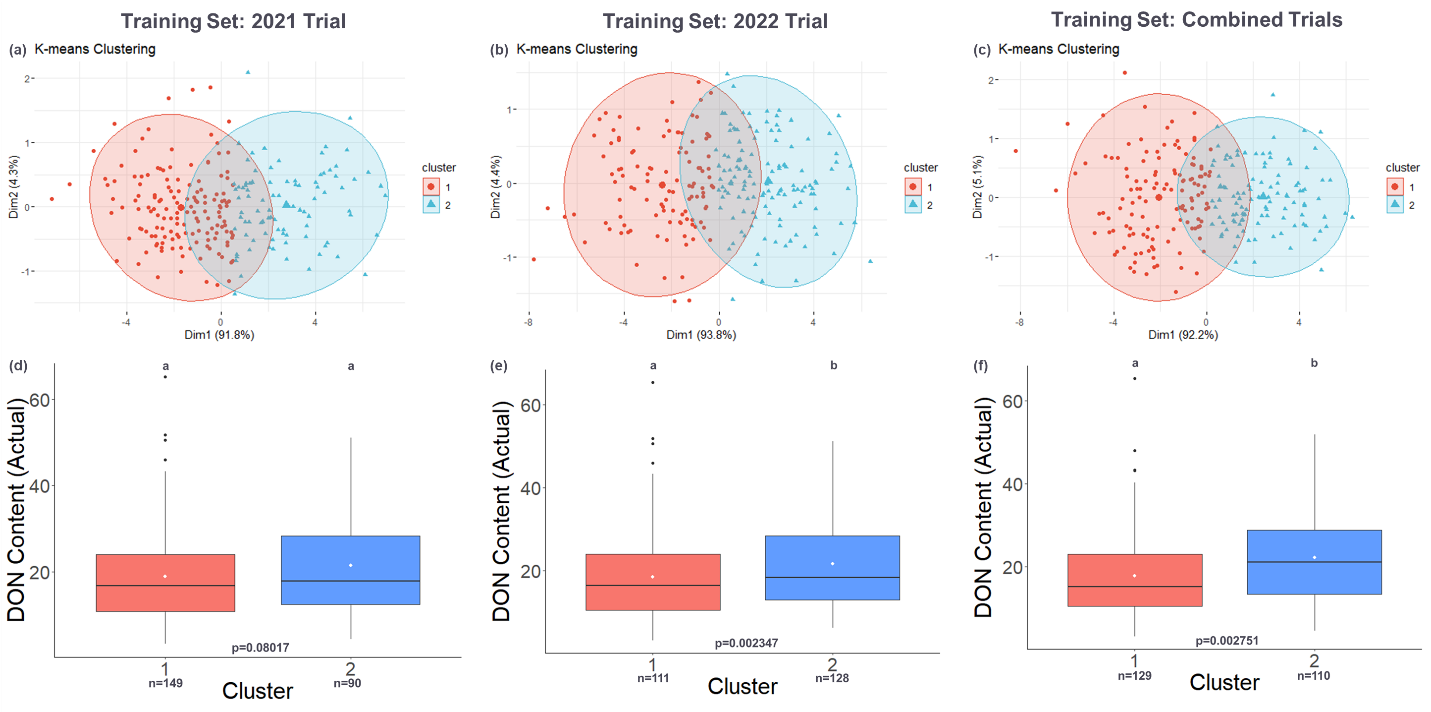


**Supplementary Figure 2.** Unsupervised K-means clustering based on blended genomic predictions of DON content in 2022 F_4:5_ selection candidates using different training sets. (a, b, c) Clustering of 2022 F_4:5_ selection candidates using genomically and predicted values. (d,e,f) Corresponding boxplots showing the actual DON content of the genotypes in Clusters 1 and 2 grouped based on phenomically and genomically predicted values. White dot represents the mean actual DON content of genotypes belonging to Clusters 1 and 2. Means with the same letter are not significantly different at alpha 0.05


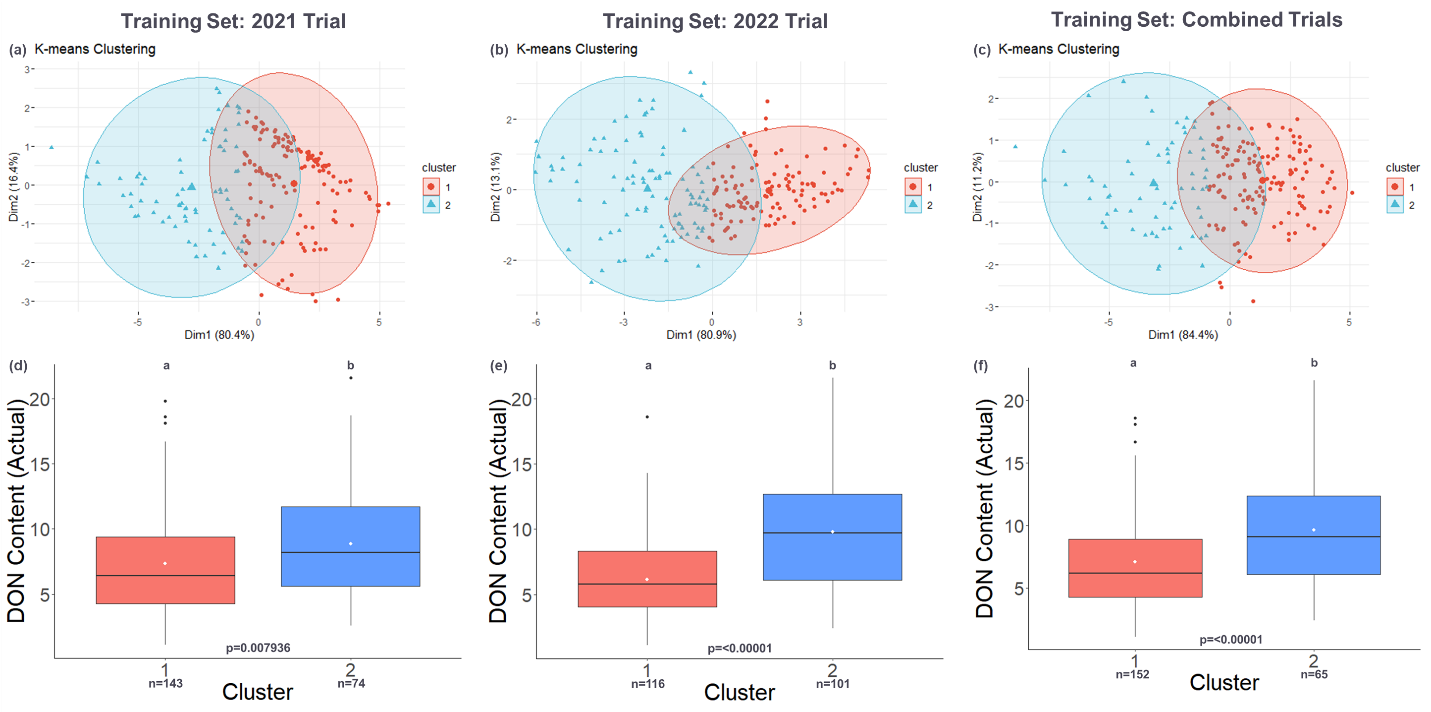


**Supplementary Figure 3.** Unsupervised K-means clustering based on blended phenomic predictions of DON content in 2023 F_4:5_ selection candidates using different training sets. (a, b, c) Clustering of 2023 F_4:5_ selection candidates using genomically and predicted values. (d,e,f) Corresponding boxplots showing the actual DON content of the genotypes in Clusters 1 and 2 grouped based on phenomically and genomically predicted values. White dot represents the mean actual DON content of genotypes belonging to Clusters 1 and 2. Means with the same letter are not significantly different at alpha 0.05


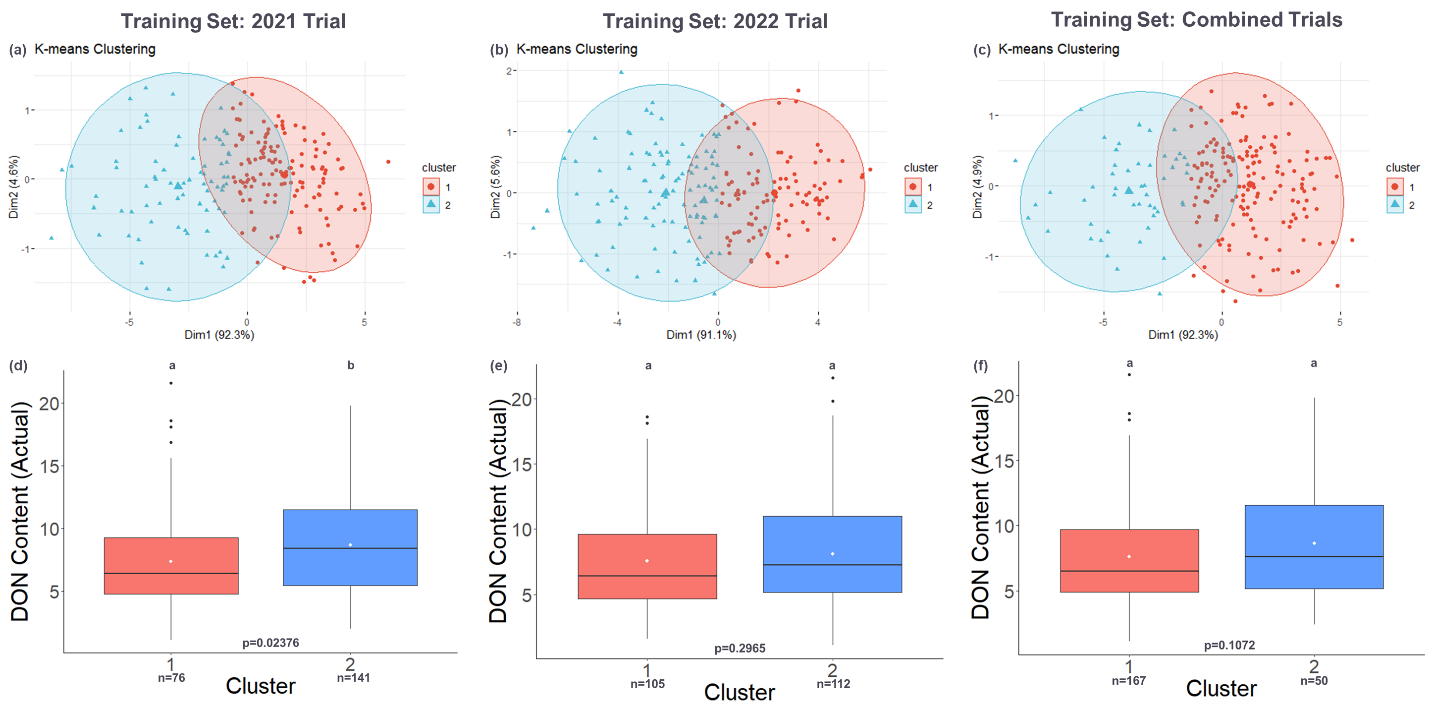


**Supplementary Figure 4.** Unsupervised K-means clustering based on blended genomic predictions of DON content in 2023 F_4:5_ selection candidates using different training sets. (a, b, c) Clustering of 2023 F_4:5_ selection candidates using genomically and predicted values. (d,e,f) Corresponding boxplots showing the actual DON content of the genotypes in Clusters 1 and 2 grouped based on phenomically and genomically predicted values. White dot represents the mean actual DON content of genotypes belonging to Clusters 1 and 2. Means with the same letter are not significantly different at alpha 0.05
